# An exploration of prenatal breastfeeding self-efficacy: a scoping review protocol

**DOI:** 10.12688/openreseurope.14938.1

**Published:** 2022-07-22

**Authors:** Liz McGovern, Aisling Geraghty, Fionnuala McAuliffe, Sharleen O'Reilly

**Affiliations:** 1 School of Agriculture and Food Science, University College Dublin, Belfield, Dublin 4, D04 V1W8, Ireland; 2 UCD Perinatal Research Centre, School of Medicine, University College Dublin, National Maternity Hospital, Dublin 2, D02 YH21, Ireland

**Keywords:** Breastfeeding, lactation, prenatal, antenatal, self-efficacy, self-belief

## Abstract

**Objective**: To synthesise the evidence on prenatal breastfeeding self-efficacy, including identifying concepts and theoretical frameworks that underpin its development, the evidence on its measurement, interventions used to improve it, and association with breastfeeding outcomes.

**Background**: Breastfeeding self-efficacy is described as a woman’s self-belief and confidence in her perceived ability to breastfeed. It is a modifiable measure that is strongly associated with breastfeeding outcomes such as initiation, exclusivity, and duration. Interventions aimed at increasing self-efficacy are often in the postnatal period and have been shown to be effective at improving breastfeeding outcomes. The prenatal period appears to be underexplored in the literature and yet focusing on enhancing it may have the potential for further improvements in self-efficacy and on subsequent breastfeeding outcomes. A comprehensive knowledge synthesis on prenatal breastfeeding self-efficacy is lacking.

**Methods**: The search will include databases across health, psychology, sociology, and the grey literature on breastfeeding guidance. Once the PCC framework (Problem: breastfeeding, Concept: self-efficacy, Context: prenatal period) is met, sources of evidence from any contextual setting will be eligible for inclusion. Limits will not be applied on geographic location or year of publication. The PRISMA-ScR flow diagram of search and study selection will be used to report final figures. Two independent reviewers will perform title and abstract screening, full-text review, and data extraction. Data will be charted to provide a logical and descriptive summary of the results that align with the objectives.

**Conclusion**: The results will provide an understanding of what has been done in the space and what gaps exist, informing recommendations for the timing of measurement and the design of prenatal interventions.

## Plain language summary

Around the world, the number of babies that are breastfed is a lot lower than the recommendation for the best possible growth, development, and health. If someone believes that they will be able to breastfeed, they have a better chance of being successful. Their belief that they can breastfeed is something that can be enhanced. Higher self-belief (self-efficacy) leads to higher breastfeeding outcomes. Many interventions to enhance breastfeeding self-efficacy happen after babies are born and breastfeeding has started. There may be a missed opportunity to focus more on increasing breastfeeding self-efficacy early in pregnancy to increase the potential improvement of this self-belief. This review will look at what is known about women’s belief in their ability to breastfeed while they are still pregnant. It will look at how and when this belief is measured and how it impacts breastfeeding. Trying to help grow this belief before the baby is born may help women start breastfeeding and breastfeed for longer.

## Introduction

The World Health Organisation (WHO) recommends exclusive breastfeeding for the first six months of life to achieve optimal growth, development and health, followed by continued breastfeeding alongside nutritious, complementary foods, up to the age of two years or beyond. Breastfeeding is also integral to the United Nation’s Sustainable Development Goals for 2030, in the areas of nutrition, health, inequity reduction and poverty reduction ^1, 2^. It is estimated that 820,000 deaths in children under 5 could be prevented annually, if all children 0–23 months old were optimally breastfed ^3^.

However, despite the benefits of breastfeeding to mothers, babies and wider society ^3^, breastfeeding rates fall short of the WHO recommendation, with only 44% of infants worldwide aged 0–6 months exclusively breastfed between 2015–2020. The World Health Assembly, as the decision-making body of the WHO, set a global nutrition target to increase exclusive breastfeeding in the first six months to 50% globally by 2025, which was revised to 70% by 2030.

Global feeding practices do vary. Zong *et al*. examined data from 57 low to middle income countries (LMICs) and concluded that breastfeeding practice improved from 2000–2018, but a big gap still exists when compared with WHO feeding recommendations, particularly for LMICs in the Eastern Mediterranean and European regions, and upper middle-income countries ^4^. Victora *et al.* found the highest rates of breastfeeding at 12 months were in sub-Saharan Africa, South Asia and part of Latin America ^3^. Most high-income countries had rates below 20% with important differences between the United Kingdom (<1%) and the United States (27%), and between Norway (35%) and Sweden (16%).

The multifaceted and complex nature of breastfeeding as a behaviour means that varying rates found between countries with similar sociocultural backgrounds exist. For example, women in Ireland and the UK are substantially less likely to breastfeed when compared with women in Australia ^5^. The rates in high income western countries vary considerably. Over a 21 years period (1984–2015) breastfeeding initiation went from 97% to 95.5% in Sweden, 57% to 82.5% in the United States and 31.7% to 58% in Ireland ^6^. Ireland has among the lowest breastfeeding rates in the world, Quinn *et al.* ^7^ reported the initiation rate in 2018 was 60%, compared with an average of 86% across OECD (Organization for Economic Co-operation and Development) countries. This increased to 63.7% in 2019 and dropped slightly to 62.3% in 2020. For most countries to achieve a meaningful increase in breastfeeding rates, strong action is required in almost all policy and programme areas ^2^. Although modest gains in exclusive breastfeeding rates have been made, the World Bank do not expect the trends to continue without investment in comprehensive strategies ^8^.

The reporting of global, national and research breastfeeding data are inconsistent. The data are historically plagued with problems of collection and comparability ^6^. The European Union reports that countries and monitoring groups appear to be applying different definitions and methods, and that WHO definitions and methods are frequently not applied. The report recommends great care is needed when making comparisons due to the lack of standardised data collection methods and in the application of definitions. In their highly cited publication on breastfeeding in the 21 ^st^ Century, Victora *et al*. ^3^ were only able to access data from 37 out of 75 high income countries (HICs), with several of these only providing a subset of information. The authors subsequently warned that the data should be interpreted with caution. The situation within Europe appears to have not improved with Zakarija-Grković *et al.*’s recent assessment of the infant feeding strategy in Europe only able to report data from 18 of the 53 WHO/EURO Member States ^9^.

Many factors, both modifiable and non-modifiable, contribute to a woman’s intention to breastfeed and their subsequent initiation and practice of breastfeeding. When supporting women to breastfeed, non-modifiable determinants include maternal age, socioeconomic status, marital status, education, geographical residence, and parity ^10– 13^. Modifiable factors include mode of delivery, previous breastfeeding experience, breastfeeding self-efficacy, attitudes to infant feeding, knowledge of the benefits of breastfeeding, and social and professional support ^5, 14– 16^. To tackle sub-optimal breastfeeding rates, Economou *et al.* ^17^ recommended that emphasis should shift to the modifiable determinants.

Breastfeeding self-efficacy has been identified as a strong modifiable predictor of breastfeeding initiation, duration, and exclusivity ^18, 19^. The term stems from Dennis’ adaptation of Bandura’s Social Cognitive Theory on self-efficacy and is described as a woman’s self-belief and confidence in her perceived ability to breastfeed ^20, 21^. There is a growing body of literature on breastfeeding intention and self-efficacy, and their influence on breastfeeding outcomes and rates. Mothers with high levels of breastfeeding self-efficacy are more likely to breastfeed exclusively for longer ^22^. It is therefore important to focus on maximising this modifiable determinant, especially in countries and cultures with relatively low breastfeeding rates.

Exploring the timing around when breastfeeding self-efficacy is developed, measured, and potentially influenced is important. Confidence and belief in the ability to breastfeed starts well before birth and Araban *et al.* reiterate the importance of there being an antenatal focus, stating that prenatal preparation was key to increasing self-efficacy ^22^. Silva-Tubio *et al.* also outline the role of prenatal breastfeeding self-efficacy in both determining groups at-risk for early cessation and in evaluating programmes that promote breastfeeding ^23^. However, much of the focus on improving self-efficacy is in the postnatal period and less attention has been paid to the prenatal one.

Breastfeeding assessment tools have been subject of systematic reviews exploring their clinical usefulness. Reviews on general tools ^24, 25^ and those specifically measuring breastfeeding self-efficacy ^26^ exist. The self-efficacy tools provide a varying number of items (5–32) with a Likert-style scale based on either agreement, confidence, or perceived ability, which are scored ^26^. The tools have been shown to be useful in measuring self-efficacy, identifying groups at higher risk of early breastfeeding cessation, exploring the changes in scores following an intervention, and in the relationship of scores with breastfeeding outcomes, such as initiation, exclusivity, and duration ^27– 29^. Specific tools exist to measure self-efficacy in the prenatal period or postnatal period ^26^ but the postnatal tools are more commonly used ^27^, regardless of the administration timepoint, potentially to facilitate prenatal and postnatal comparisons following intervention. The timing of prenatal interventions and assessment are often in the third trimester, commonly 36–38 weeks gestation ^30^, rather than early in pregnancy. This may be a missed opportunity to focus on influencing self-efficacy scores over a longer period in pregnancy.

Social psychology can help to better understand the relationship between psychosocial determinants of breastfeeding self-efficacy and breastfeeding outcomes. The most frequently used theories have been the Theory of Reasoned Action, Theory of Planned Behavior, and the Breastfeeding Self Efficacy Framework ^31^. Theoretical approaches, such as these, inform the content design of both tools used to assess breastfeeding self-efficacy and the interventions designed to impact self-efficacy scores and breastfeeding outcomes. The theoretical framework used to inform the design of four of the six breastfeeding self-efficacy assessment tools ^18, 32– 34^ was Bandura’s Social Cognitive Theory ^20, 21^. This theory explains how people choose and maintain health behaviour, with the belief that they will attempt what they believe they can do. For example, the Breastfeeding Self-Efficacy Scale (BSES) has items on three factors: breastfeeding technique, intrapersonal thoughts, and support ^18^. Some tools designed to measure prenatal breastfeeding self-efficacy ^35, 36^ are based on the theory of planned behaviour and theory of reasoned action. They stem from Ajzen’s guiding belief that people’s intentions are strongly linked to their actual behaviour ^37^. For example, the Prenatal Rating of Efficacy in Preparation to Breastfeed Scale (PREP to BF Scale) has items on four factors: individual processes, interpersonal processes, professional advice, and social support ^36^.

Interventions aimed at improving breastfeeding self-efficacy often focus on educational elements, which may not reflect the breadth of factors in the theories that underpin it. Systematic reviews examining interventions targeting breastfeeding self-efficacy and breastfeeding outcomes focused on education ^27, 29, 38^, with just one review having a wider scope that included education and support ^30^. Maleki *et al.* observed that theory-based education led to greater improvement in self-efficacy scores compared with non-theory-based education ^29^. Chipojola *et al.* ^38^ found that the theoretical approach used, impacted breastfeeding outcomes in different ways. In their systematic review and meta-analysis of theory-based educational interventions on breastfeeding self-efficacy and exclusive breastfeeding, breastfeeding self-efficacy scores at 1–2 months postpartum were improved when educational interventions guided by the breastfeeding self-efficacy framework were used, which was consistent with the findings from another systematic review conducted by Brockway *et al.* ^27^. The educational interventions that used planned behaviour theory were more likely to lead to higher exclusive breastfeeding rates at 3–6 months. They recommended that future educational interventions to improve breastfeeding outcomes consider integrating aspects from both theoretical models into the design of their intervention ^38^.

This review will explore breastfeeding self-efficacy in the prenatal period. It is a modifiable measure that is strongly associated with breastfeeding outcomes such as initiation, exclusivity, and duration. Interventions aimed at increasing this measure in antenatal and postnatal care have been effective at improving breastfeeding outcomes ^27^. However, breastfeeding self-efficacy in the prenatal period appears to be underserved in the literature. The use of instruments and interventions in the prenatal period and their theoretical foundation will be explored. Enhancing breastfeeding self-efficacy antenatally may have the potential for greater improvements in subsequent breastfeeding outcomes. A comprehensive knowledge synthesis on breastfeeding self-efficacy in the prenatal period is needed.

### Aims & objectives

Aim: To explore and synthesise the current literature and evidence base on prenatal breastfeeding self-efficacy.

Objectives:

- Identify and describe the theoretical frameworks and influencing factors that underpin prenatal breastfeeding self-efficacy.
- Identify and describe the evidence on the measurement of prenatal breastfeeding self-efficacy.
- Identify and describe the interventions used to improve prenatal breastfeeding self-efficacy and their impact on breastfeeding outcomes (initiation, duration, and exclusivity).

## Methods

### Study design

A scoping review was determined to be the most appropriate methodology to address the broad and holistic exploration needed. It facilitates the systematic mapping of the literature available, identifying the extent and nature of key concepts, theories, sources and types of evidence, and potential gaps in research. The review will be conducted in accordance with the Joanna Briggs Institute (JBI) approach for the conduct of scoping reviews, which was informed by Arksey & O’Malley ^39^ and Levac *et al.* ^40^. The Preferred Reporting Items for Systematic Reviews and Meta-Analysis extension for Scoping Reviews (PRISMA-ScR) checklist will be used in designing, reviewing, and reporting this review ^41^. The scoping review is registered with Open Science Framework ( 10.17605/OSF.IO/U85WK).

### Review team

The review will be conducted by a team that consists of an International Board-Certified Lactation Consultant - IBCLC (LMcG), registered midwife (LOT), registered dietitian (SOR) and obstetrician (FMcA). The team reflects the range of health professionals engaged in providing antenatal care to women for pregnancy and breastfeeding.

### Eligibility criteria

Eligibility criteria will follow the PCC framework, using ‘problem’ for P rather than the more commonly used ‘population’. The PCC framework (Problem, Concept, Context) will be used to define eligibility alongside additional inclusion and exclusion criteria ( Table 1). A preliminary search conducted of MEDLINE, the Cochrane Database of Systematic Reviews, PROSPERO and the JBI Evidence Synthesis database did not identify any existing or registered reviews on the topic. Any existing or registered breastfeeding self-efficacy reviews focused on the postnatal period or had a narrow focus on specific intervention types.

**Table 1.** Eligibility criteria incorporating the (PCC) framework - Problem, Concept, Context.

Eligibility criteria	Description
Problem: Breastfeeding	It is important to be as inclusive as possible so no limits will be applied for this criterion. The review will include any literature with reference to: • Mode of breastfeeding - feeding at the breast, expressing milk by hand or pump, or using assistive devices like nipple shields or supplemental feeding systems • Extent of breastfeeding - exclusively supplying their baby’s needs or partial breastfeeding along with infant formula • Experience of breastfeeding - whether first or subsequent baby regardless of previous experience or duration of breastfeeding.
Concept: Self-Efficacy	The review will include literature related to breastfeeding self-efficacy and exclude those focusing on self-efficacy of other parenting areas. To avoid unintentional exclusion of relevant results that have not used ‘breastfeeding self-efficacy’ terminology, the search strategy will incorporate the use of additional terminology related to the concept and its development, for example: self-belief, confidence, motivation, and intention to breastfeed.
Context: Prenatal Period	The review will consider results for inclusion: • Focusing on breastfeeding self-efficacy before birth, including pre-pregnancy • Spanning the prenatal and postnatal periods, where data is provided for the prenatal period, even if the focus of the review is a postnatal intervention.
Language	A limit of English language will be applied, due to time constraints.
Date range	The search will not be limited by date range.
Study design	This scoping review will include sources of research evidence from any contextual setting, once each source meets the inclusion criteria. • Experimental / quasi-experimental designs including randomised and non-randomised controlled trials, before and after studies and interrupted time-series studies • Analytical observational studies including prospective and retrospective cohort studies, case-control studies, and analytical cross-sectional studies • Descriptive observational study designs including case series, case reports and descriptive cross-sectional studies • Qualitative studies • Systematic reviews that meet the inclusion criteria, depending on the research question

### Search strategy

Published and unpublished studies and literature will be sourced across a variety of disciplines including health, psychology, and sociology. To support the development of the search strategy, an initial, limited search of MEDLINE and EMBASE was undertaken to identify articles on the topic. The terminology used in the titles, abstracts and index terms of relevant articles was used to supplement an initial list of keywords, adding synonyms and variations where appropriate, for the sample search strategy provided in Table 2. The final search strategy, including all identified keywords and index terms, will be adapted for each database and/or information source to be searched, incorporating the database thesaurus / directory terms where applicable.

**Table 2.** Sample Search Strategy in EMBASE database.

	Search Terms & Commands
1	(breastfe* OR ‘breast fe*’ OR lactat*):ti:ab OR ('breastfeeding'/exp/mj OR 'lactation'/exp/mj)
	**AND**
2	(‘self efficacy' OR ‘self-efficacy' OR efficacy OR ‘confiden*’ OR motivat* OR belief* OR ‘inten* to breastfeed’ OR ‘breastfeed* intentioVn*’):ti:ab OR ('efficacy'/exp/mj OR 'confidence'/exp/mj OR 'motivation'/exp/mj)
	**AND**
3	(prenatal* OR antenatal* OR 'ante-natal*' OR ‘pregnan*’):ti:ab OR ('prenatal'/exp/mj OR 'prenatal care'/exp/mj OR 'prenatal period'/exp/mj OR 'pregnant woman'/exp/mj OR 'pregnancy'/exp/mj)

The databases to be searched are Medline (Ovid), Cumulative Index to Nursing and Allied Health Literature (CINAHL), EMBASE, PsycINFO, Global Health, International Bibliography of Social Sciences (IBSS), Applied Social Science Index and Abstracts (ASSIA) and Web of Science. A variety of grey literature sources will be searched to identify relevant content to be considered for inclusion, such as reviews, reports, and conference proceedings. A focused Google search will be performed alongside website searches of relevant international organisations that have policy, advisory or guidance roles in breastfeeding, including the World Health Organisation (WHO), United Nations International Children’s Emergency Fund (UNICEF), the Global Breastfeeding Collective, the Academy of Breastfeeding Medicine, and La Leche League International. Theses and dissertations will also be considered for inclusion through searching the ProQuest Dissertation & Theses Global (PQDT) database.

Backward and forward snowballing will be used to source additional potential studies for inclusion. The procedure for backward snowballing will be through the screening of included article’s reference lists. The forward snowballing will be through identification of new papers citing the included articles. Authors of primary studies may be contacted for further information, if required.

### Study / Source of evidence selection

The citations identified will be exported and uploaded into Covidence (Veritas Health Innovation, Melb, AU) where duplicates will be removed. Screening consistency pilot tests will be conducted at both the title and abstract, and full text screening stages, where a minimum of 90% agreement is required between reviewers before progressing. Title and abstract screening, followed by full texts retrieval and screening, will be independently conducted by at least two reviewers using the inclusion criteria. Any disagreements between reviewers at either stage of the selection process will be resolved through discussion, or with an additional, independent reviewer.

The search results and study inclusion process, including the reasons for exclusion, will be reported in full using the Preferred Reporting Items for Systematic Reviews and Meta-Analyses extension for Scoping Review (PRISMA-ScR) flow diagram ^41^.

### Data extraction

The data will be extracted using different extraction forms that will be developed by the reviewers in line with inclusion criteria related to each objective ( Table 3). Some sources will have data extracted for more than one objective of the scoping review. The data will be extracted by at least two independent reviewers, following a pilot test of the process. The extraction of data on study characteristics and findings for primary studies will follow standard systematic review procedures. Similarly, data in systematic or scoping reviews will follow the same extraction process.

**Table 3.** Data extraction criteria for each objective.

Objective 1:	Objective 2:	Objective 3:
*Identify and describe the theoretical* *frameworks and influencing factors that* *underpin the development of prenatal* *breastfeeding self-efficacy*	*Identify and describe the evidence* *on the measurement of prenatal* *breastfeeding self-efficacy*	*Identify and describe the interventions* *used to improve prenatal breastfeeding* *self-efficacy and their impact on outcomes*
Any of the following:	Any of the following:	Any of the following:
• Theoretical frameworks used in instruments to assess or interventions to influence prenatal breastfeeding self-efficacy	• Tools used to measure prenatal breastfeeding self-efficacy	• All levels of intervention to change prenatal breastfeeding self-efficacy, not limited to randomised controlled trials
• Theoretical frameworks referred to re: development of prenatal breastfeeding self-efficacy	• Measurements other than tools for prenatal breastfeeding self-efficacy	• Timing - description of the time periods involved in the interventions measuring prenatal self-efficacy, including those with postnatal interventions
• Discussion of factors that underpin, influence, or predict prenatal breastfeeding self-efficacy	• Content, format, use, timing, reliability and validity of instruments for prenatal breastfeeding self-efficacy	• Description of the intervention, delivery agent, setting, context, population, duration, effectiveness, and any other relevant study data

The data extraction for grey literature will follow a more qualitative approach, extracting themes and quotes from sources. To maintain standards of transparency, each reviewer will keep notes on any areas of discrepancies or ambiguous descriptions to support resolution of conflicts.

The data extraction will be an iterative process and the data extraction tools may be modified and revised during the process. Any such modifications will be detailed in the final scoping review. Disagreements that arise between the reviewers will be resolved through discussion, or with an additional independent reviewer. If appropriate, authors of papers will be contacted to request missing or additional data, where required.

A draft extraction form is provided in Table 4. A critical appraisal of the evidence will not be conducted as this is beyond the purpose of a scoping review. In its place, the reviewers will assess what level of evidence each individual source comes from against the JBI categories. This will give the scoping review the ability to provide comment on the quality of the evidence informing the state of understanding.

**Table 4.** Sample Data Extraction Form - Objective 2: Measurement of Breastfeeding Self Efficacy.

		Details
Title		
Author		
Journal / Reference			
DOI		
Measurement Method	Tool:	
	Other:	Content: Format:
Timing of measurement		
Reliability & Validity		
Method of administration		
Sample characteristics		
Outcome Measure		
Key Findings		
Conclusions		
Recommendations		

### Data analysis and presentation

The analysis will focus on mapping the data from the data extraction templates against the review objectives. The primary study data will be presented describing the study characteristics and an exploratory narrative. The grey literature sources will be described as fully as possible, and an exploratory narrative will be used. The analysis of concepts and theories underpinning prenatal breastfeeding self-efficacy and synthesising the evidence available on its measurement and outcomes will be subject to the level and quality of data identified. The data will be presented in a tabular or diagrammatic form that most appropriately aligns to the objectives. The tables and charts will report on publication details and data specific to each of the objectives. Qualitative data will be content analysed using NVivo version 12, where appropriate (a free open source alternative is Taugette). A narrative summary will accompany the results and will describe how they relate to the overall scope of the review.

### Dissemination

The results of the review will be disseminated in a variety of ways. The primary planned outcome will be the full scoping review publication. The secondary planned outcome will be presentations at the Association of Lactation Consultants of Ireland and other international conferences. The third planned outcome would be blog and website posts developed for the EU Horizon2020 funded IMPACT DIABETES B2B project to potentially reach wider stakeholders including women and their partners. The final planned dissemination outcome, if appropriate, will be a policy brief for the relevant health service national breastfeeding coordinators in IMPACT DIABETES B2B partner countries.

## Conclusion / discussion

This scoping review will provide an important synthesis of the literature on prenatal breastfeeding self-efficacy and develop our understanding of what gaps exist. The review will advance our knowledge of the theoretical basis underpinning prenatal breastfeeding self-efficacy, whether it has been applied to date and what gaps exist in any interventional settings. The review will also potentially inform recommendations on the timing and methods of prenatal breastfeeding self-efficacy assessment and the design of effective prenatal interventions. In summary, this scoping review will make an important contribution to advancing whether prenatal breastfeeding self-efficacy has a sufficient evidence base to support delivering improvements in breastfeeding outcomes.

## Data availability statement

No data are associated with this article.
