# Supplementary material for: An exploration of prenatal breastfeeding self-efficacy: a scoping review protocol
Source: Open Res Eur. 2023 Jan 24;2:91. Originally published 2022 Jul 22. [Version 3] doi: 10.12688/openreseurope.14938.3 (PMC10445816; doi:10.12688/openreseurope.14938.3)
Supplement: Peer review history

# Peer review: Reviewer response for version 3

**DOI:** 10.21956/openreseurope.16800.r30706

**Published:** 2023-01-27

**Authors:** Kathleen Kendall-Tackett

**Affiliations:** 1 Department of Pediatrics, School of Medicine, Texas Tech University Health Sciences Center, Amarillo, TX, USA

Is the study design appropriate for the research question?

Yes

Is the rationale for, and objectives of, the study clearly described?

Yes

Are sufficient details of the methods provided to allow replication by others?

Partly

Are the datasets clearly presented in a useable and accessible format?

Not applicable

Reviewer Expertise:

NA

I confirm that I have read this submission and believe that I have an appropriate level of expertise to confirm that it is of an acceptable scientific standard.

# Peer review: Reviewer response for version 2

**DOI:** 10.21956/openreseurope.16428.r30284

**Published:** 2022-12-14

**Authors:** Kathleen Kendall-Tackett

**Affiliations:** 1 Department of Pediatrics, School of Medicine, Texas Tech University Health Sciences Center, Amarillo, TX, USA

I feel that the author has done a great job of addressing my original comments. My only remaining comment is on the choice of search terms on Table 2. The author is going to search using not only breastfeeding, but chestfeeding, and human-milk feeding. I have several concerns with that strategy.

1. Where I suspect the literature is sparse on prenatal breastfeeding self-efficacy, I expect that it will be non-existent for prenatal chestfeeding and human-milk feeding.
2. And this is the larger concern. Every, single reference the author has included is on breastfeeding. There is not a single reference for the other two terms. These behaviors are quite different from each other. Does the BF self-efficacy scale even apply to *prenatal *chestfeeding or human milk feeding? **Who plans to do that prenatally? We don't know. **Many women who human-milk feed often did not plan to do that prenatally but often encountered an obstacle (or more than one) that stopped them from feeding at the breast. And what of chestfeeding? That can represent everything from just renaming breastfeeding to chestfeeding to having top surgery and feeding with a supplementer. Those are very different behaviors. Does that scale accurately measure it? We don't know. If the author is including these other terms, there needs to be rationale provided in her introduction. Yet, there's no mention of it at all. That is a significant oversight, in my view, that the author needs to address before this can move forward.

Is the study design appropriate for the research question?

Yes

Is the rationale for, and objectives of, the study clearly described?

Yes

Are sufficient details of the methods provided to allow replication by others?

Partly

Are the datasets clearly presented in a useable and accessible format?

Not applicable

Reviewer Expertise:

NA

I confirm that I have read this submission and believe that I have an appropriate level of expertise to confirm that it is of an acceptable scientific standard, however I have significant reservations, as outlined above.

# Response

**Published:** 2023-01-19

**Authors:** Liz McGovern

**Affiliations:** University College Dublin, Ireland

***"I feel that the author has done a great job of addressing my original comments."***

*Response* *: We thank the reviewer for this positive feedback. *  

***"My only remaining comment is on the choice of search terms on Table 2. The author is going to search using not only breastfeeding, but chestfeeding, and human-milk feeding. I have several concerns with that strategy. 1. Where I suspect the literature is sparse on prenatal breastfeeding self-efficacy, I expect that it will be non-existent for prenatal chestfeeding and human-milk feeding.  2. And this is the larger concern. Every, single reference the author has included is on breastfeeding. There is not a single reference for the other two terms. These behaviors are quite different from each other. Does the BF self-efficacy scale even apply to prenatal chestfeeding or human milk feeding? Who plans to do that prenatally? We don't know. Many women who human-milk feed often did not plan to do that prenatally but often encountered an obstacle (or more than one) that stopped them from feeding at the breast. And what of chestfeeding? That can represent everything from just renaming breastfeeding to chestfeeding to having top surgery and feeding with a supplementer. Those are very different behaviors. Does that scale accurately measure it? We don't know. If the author is including these other terms, there needs to be rationale provided in her introduction. Yet, there's no mention of it at all. That is a significant oversight, in my view, that the author needs to address before this can move forward."***

*Response: We thank the reviewer for raising these concerns in points 1 and 2. The terms ‘chestfeeding’ and ‘human milk feeding’ were added to the search strategy in response to a peer reviewer recommendation for term inclusivity. We agree that it is very unlikely in a field where there is a dearth of evidence generally, that there will be many results focusing on these specific circumstances, but recognise the importance of their inclusion, particularly within a scoping review methodology. We acknowledge that the self-efficacy scales are general and may not yet have been applied or may not apply to specific circumstances. It is possible that a study may explore whether parents with higher prenatal breastfeeding self-efficacy scores, using the assessment tools available or another measure of breastfeeding confidence, are more likely to provide human milk (expressed or donor) rather than infant formula, or to chestfeed for whatever reason. Such a study may exclusively use the included terms instead of breastfeeding. We remain convinced that an inclusive definition of feeding practices will benefit this scoping review. We thank the reviewer for highlighting the need for clarification around the inclusion of these terms in the sample search strategy in Table 2. We have added the following paragraph to the inclusion criteria within the methods section:*

 In recognition of the move towards more inclusive language in the field of lactation, the terms ‘chestfeeding’ and ‘human milk feeding’ are included in the search strategy under the breastfeeding criteria [refs]. Both terms may be used by non-binary parents and those caring for them regarding their infant feeding, and chestfeeding may also refer to the use of donor milk or infant formula via a supplementer following transgender top surgery. For this scoping review an inclusive definition of breastfeeding will be applied within the search terminology and inclusion criteria.

**References:** Dinour LM. Speaking Out on "Breastfeeding" Terminology: Recommendations for Gender-Inclusive Language in Research and Reporting. Breastfeed Med. 2019 Oct;14(8):523-532. doi: 10.1089/bfm.2019.0110 MacDonald TK. Lactation Care for Transgender and Non-Binary Patients: Empowering Clients and Avoiding Aversives. J Hum Lact. 2019 May;35(2):223-226. doi: 10.1177/0890334419830989

*The breastfeeding inclusion criterion in Table 1 has also been modified for clarity as follows:*

**Breastfeeding** It is important to be as inclusive as possible so no limits will be applied for this criterion. The review will include any literature with reference to:

- Mode of breastfeeding - feeding at the breast, expressing milk by hand or pump, or using assistive devices like nipple shields or supplemental feeding systems. This includes the use of the terms chestfeeding and human milk feeding for inclusivity.
- Extent of breastfeeding - exclusively supplying their baby’s needs or partial breastfeeding along with infant formula.
- Experience of breastfeeding - whether first or subsequent baby regardless of previous experience or duration of breastfeeding.

# Peer review: Reviewer response for version 2

**DOI:** 10.21956/openreseurope.16428.r30283

**Published:** 2022-12-08

**Authors:** Lyndsey Hookway

**Affiliations:** 1 School of Health and Social Care, Swansea University, Swansea, UK

Thank you for the revisions. I feel that the protocol now has more clarity of purpose and scope and more clearly outlines the goal of the review. The wording in the introduction and the explanation of the timing of self-efficacy interventions is much better.

 Thank you for addressing the minor methodological inconsistencies relating to the use and implementation of the final review to inform practice in terms of scoping vs systematic review aims and purpose. I feel this is now clearer for the reader to understand the possible contribution the findings will have.

 I look forward to reading the review.

Is the study design appropriate for the research question?

Partly

Is the rationale for, and objectives of, the study clearly described?

Yes

Are sufficient details of the methods provided to allow replication by others?

Partly

Are the datasets clearly presented in a useable and accessible format?

Not applicable

Reviewer Expertise:

Lactation, children's health, paediatrics, medical complexity breastfeeding, sleep, responsive parenting

I confirm that I have read this submission and believe that I have an appropriate level of expertise to confirm that it is of an acceptable scientific standard.

# Peer review: Reviewer response for version 1

**DOI:** 10.21956/openreseurope.16142.r29936

**Published:** 2022-09-05

**Authors:** Lyndsey Hookway

**Affiliations:** 1 School of Health and Social Care, Swansea University, Swansea, UK

The authors propose a scoping review which has been well-designed with clear objectives and transparent process. I particularly note the acknowledgement of very low breastfeeding rates in the UK and Ireland and wonder whether the review will consider geographical disparities both in the implementation and also outcomes of self-efficacy promoting strategies. Obviously, rates of breastfeeding and the extent to which breastfeeding motivation may be modifiable by self-efficacy interventions are likely to vary. Will there be some discussion about geographically/culturally/contextually different reasons for differing breastfeeding rates? My concern is that it may be difficult to distinguish the other confounding factors to isolate self-efficacy interventions as the reason for improved breastfeeding rates. Essentially - does the intervention always have the same outcome in all populations, or will there be some discussion of which communities may be essentially 'lower hanging fruit'? And to what extent will other factors influence breastfeeding rates above and beyond self-efficacy measures?

 The authors also note that most self-efficacy strategies are implemented postnatally and postulate that antenatally implemented strategies may be more effective. Is this an assumption, or will there be some attempt to compare the efficacy of different timings? This part of the review could perhaps be clearer. If one of the aims is to elucidate the effectiveness of prenatal versus postnatal self-efficacy work it feels important to clarify whether there is a demonstrable improvement on breastfeeding outcome with prenatal intervention versus postnatal. 

 One minor suggestion is to include the term chestfeeding and human milk feeding to the search terms for completeness.

 Finally, at times, this scoping review reads like a protocol for a systematic review. I feel like the distinction between systematic reviews and scoping reviews could be clearer, with perhaps some theoretical justification for the choice of a scoping review versus a systematic review. For example, in the conclusion, the authors state that the review will establish whether prenatal self-efficacy has a sufficient evidence base to support this intervention. Systematic reviews are more commonly used to inform the development of clinical recommendations, and assess whether a particular practice is sufficiently evidence based. If this is a scoping review, then determining whether the evidence for prenatal intervention is of sufficient quality to justify its use may be beyond the commonly recommended indications for this type of review (particularly if you are using grey literature without clear protocols for whether you will be using CASP criteria to assess their rigour). It sounds in places like the methodology has become slightly mixed. I like Munn *et al* (2018) for a very clear explanation of the differences. ^1^

 Overall, this is an interesting area of breastfeeding research and could have the potential to improve our delivery of antenatal care, especially to vulnerable populations with very low breastfeeding rates. I look forward to reading it.

Is the study design appropriate for the research question?

Partly

Is the rationale for, and objectives of, the study clearly described?

Yes

Are sufficient details of the methods provided to allow replication by others?

Partly

Are the datasets clearly presented in a useable and accessible format?

Not applicable

Reviewer Expertise:

Lactation, children's health, paediatrics, medical complexity breastfeeding, sleep, responsive parenting

I confirm that I have read this submission and believe that I have an appropriate level of expertise to confirm that it is of an acceptable scientific standard, however I have significant reservations, as outlined above.

## References

[1] : Systematic review or scoping review? Guidance for authors when choosing between a systematic or scoping review approach. *BMC Medical Research Methodology* .2018;18(1) : 10.1186/s12874-018-0611-x 10.1186/s12874-018-0611-x 30453902PMC6245623

# Response

**Published:** 2022-10-06

**Authors:** Liz McGovern

**Affiliations:** University College Dublin, Ireland

We would like to thank you for your time and expert peer review of our manuscript. We appreciate your feedback and feel it has improved the quality of the protocol. We have provided a point-by-point response (in italics) indicating how we addressed each concern raised by each reviewer and the changes made. The changes are approved by all authors and highlighted in bold in addition to being tracked changes in the accompanying manuscript.  

Reviewer Comment: The authors propose a scoping review which has been well-designed with clear objectives and transparent process.

*Response: We thank the reviewer for their positive feedback.*

Reviewer Comment: I particularly note the acknowledgement of very low breastfeeding rates in the UK and Ireland and wonder whether the review will consider geographical disparities both in the implementation and also outcomes of self-efficacy promoting strategies. Obviously, rates of breastfeeding and the extent to which breastfeeding motivation may be modifiable by self-efficacy interventions are likely to vary. Will there be some discussion about geographically/culturally/contextually different reasons for differing breastfeeding rates? My concern is that it may be difficult to distinguish the other confounding factors to isolate self-efficacy interventions as the reason for improved breastfeeding rates. Essentially - does the intervention always have the same outcome in all populations, or will there be some discussion of which communities may be essentially 'lower hanging fruit'? And to what extent will other factors influence breastfeeding rates above and beyond self-efficacy measures?

*Response: We agree with the reviewer that these are very valid considerations within the area of breastfeeding rates and interventions to improve self-efficacy. This scoping review will explore the current evidence on prenatal breastfeeding self-efficacy and although we will potentially be able to explore some geographical and/or cultural aspects, this will be contingent on the data identified. The scoping review seeks to identify and map evidence in relation theories used, measurements of self-efficacy, interventions used and stated outcomes. Ideally this will help inform future work on the topic.*

Reviewer Comment: The authors also note that most self-efficacy strategies are implemented postnatally and postulate that antenatally implemented strategies may be more effective. Is this an assumption, or will there be some attempt to compare the efficacy of different timings? This part of the review could perhaps be clearer. If one of the aims is to elucidate the effectiveness of prenatal versus postnatal self-efficacy work it feels important to clarify whether there is a demonstrable improvement on breastfeeding outcome with prenatal intervention versus postnatal.

*Response: Our postulation about the effectiveness of enhancing antenatal self-efficacy forms part of our rationale of conducting the scoping review. The postnatal period is beyond the scope of our review so no comparison of these timepoints will be attempted. If sufficient data is identified, it may be possible to explore different timepoints across the prenatal period but this is not one of our objectives. We aim to describe the work to date in the area, which may inform future work to allow such a comparison. We have changed the wording at the end of the introduction section to improve the clarity of this:* “ **This review will explore breastfeeding self-efficacy in the prenatal period. It is a modifiable measure that is strongly associated with breastfeeding outcomes such as initiation, exclusivity, and duration. Interventions aimed at increasing this measure in antenatal and postnatal care have been effective at improving breastfeeding outcomes 27 . However, breastfeeding self-efficacy in the prenatal period appears to be underserved in the literature.”**

Reviewer comment: One minor suggestion is to include the term chestfeeding and human milk feeding to the search terms for completeness.

*Response: We thank the reviewer for these suggestions and agree they are valuable terms to include for completeness. We have incorporated them into the search strategy.* “(breastfe* OR ‘breast fe*’ OR lactat* OR chestfeed* OR chestfed OR 'chest feed*' OR 'chest fed' OR ' human-milk-feed*' OR 'human-milk-fed'):ti:ab”

Reviewer comment: Finally, at times, this scoping review reads like a protocol for a systematic review. I feel like the distinction between systematic reviews and scoping reviews could be clearer, with perhaps some theoretical justification for the choice of a scoping review versus a systematic review. For example, in the conclusion, the authors state that the review will establish whether prenatal self-efficacy has a sufficient evidence base to support this intervention. Systematic reviews are more commonly used to inform the development of clinical recommendations, and assess whether a particular practice is sufficiently evidence based. If this is a scoping review, then determining whether the evidence for prenatal intervention is of sufficient quality to justify its use may be beyond the commonly recommended indications for this type of review (particularly if you are using grey literature without clear protocols for whether you will be using CASP criteria to assess their rigour). It sounds in places like the methodology has become slightly mixed. I like Munn et al (2018) for a very clear explanation of the differences.

*Response: We thank the reviewer for this valuable feedback. We do state our justification for conducting a scoping review in the methods section:* **“A scoping review was determined to be the most appropriate methodology to address the broad and holistic exploration needed. It facilitates the systematic mapping of the literature available, identifying the extent and nature of key concepts, theories, sources and types of evidence, and potential gaps in research.”** *However, we agree that parts of the conclusion section veer into the area of implications for practice, which is outside the scope of a scoping review. We thank you for highlighting this minor methodological inconsistency. To address this, we have made the following changes to the conclusion section:* **“The review will also potentially inform recommendations and support future research on the timing and methods of assessing prenatal breastfeeding self-efficacy assessment and the design of effective prenatal interventions. In summary, this scoping review will make an important contribution to advancing whether our knowledge of prenatal breastfeeding self-efficacy has a sufficient evidence base and it’s potential to support delivering improvements in breastfeeding outcomes.”** *In response to feedback from both reviewers on our treatment of grey literature and assessment of quality of evidence, we have removed the reference to assessing the level of evidence against the JBI categories in the final paragraph of the data extraction section (see below). We will not be seeking to evaluate the quality of the evidence as this aligns with the focus of a systematic review and we do not yet have the knowledge of the extent of the literature to undertake such work, hence our need for a scoping review. We thank the reviewers for highlighting our methodological inconsistency and feel this change aligns the manuscript more fully with scoping review methods.* **“A draft extraction form is provided in Table 4. A critical appraisal of the evidence will not be conducted as this is beyond the purpose of a scoping review. In its place, the reviewers will assess what level of evidence each individual source comes from against the JBI categories. This will give the scoping review the ability to provide comment on the quality of the evidence informing the state of understanding.”** *To address the rigour of study selection and data extraction, a systematic approach is being adopted, including all stages of screening and data extraction conducted independently by at least 2 authors. The characteristics of any grey literature included in the review will be described. However, we agree that more clarity would be helpful and we have amended the text in the data extraction section as follows:* **“The data extraction for grey literature will depend on the nature of the data, following the methods outlined above when applicable and following a qualitative approach when required, extracting themes and quotes from sources.”**

Reviewer Comment: Overall, this is an interesting area of breastfeeding research and could have the potential to improve our delivery of antenatal care, especially to vulnerable populations with very low breastfeeding rates. I look forward to reading it.

*Response: We thank the reviewer for their positive feedback and enthusiasm.*

# Peer review: Reviewer response for version 1

**DOI:** 10.21956/openreseurope.16142.r29765

**Published:** 2022-08-25

**Authors:** Kathleen Kendall-Tackett

**Affiliations:** 1 Department of Pediatrics, School of Medicine, Texas Tech University Health Sciences Center, Amarillo, TX, USA

In this manuscript, the authors propose a systematic review of the literature on breastfeeding self-efficacy during pregnancy. Overall, the authors have been clear about their goals.

 One suggestion is to expand the section on self-efficacy. Tell readers how it influences health behavior and is super relevant to many aspects of physical and mental health. Then leap to Dennis' adaptation of it to breastfeeding. ^1^ I've always thought that it was relevant. 

 I'm not sure if you want to include this, but so much of the literature is about prenatal attachment and breastfeeding. The authors might want to distinguish between the two and offer justification for why they are focusing on self-efficacy.

 Are there a lot of articles looking at self-efficacy in early pregnancy? I would think that that is a fairly novel idea. Would there be enough literature to compare early vs late prenatal self-efficacy?

 Is the aim to "explore the theoretical underpining" of self-efficacy? That seems different than what was described in the intro. What constructs would the authors explore? It might be good to highlight those if the authors have some ideas about what they are.

 The authors forgot to add "lactation" to their list of data sources. They will find a lot there.

 PubMed is not included in the search? That seems like a key source. It would be good to include it.

 I am a bit concerned that much of the "grey literature" would not have the rigor required to be included. Will this literature be subjected to the same criteria or will it be different? 

 I'm looking forward to seeing the final review. I think it will be helpful.

Is the study design appropriate for the research question?

Yes

Is the rationale for, and objectives of, the study clearly described?

Yes

Are sufficient details of the methods provided to allow replication by others?

Partly

Are the datasets clearly presented in a useable and accessible format?

Not applicable

Reviewer Expertise:

Health psychology, lactation, perinatal mental health, trauma, women's health, breastfeeding

I confirm that I have read this submission and believe that I have an appropriate level of expertise to confirm that it is of an acceptable scientific standard, however I have significant reservations, as outlined above.

## References

[1] : Theoretical underpinnings of breastfeeding confidence: a self-efficacy framework. *J Hum Lact* .1999;15(3) : 10.1177/089033449901500303 195-201 10.1177/089033449901500303 10578797

# Response

**Published:** 2022-10-06

**Authors:** Liz McGovern

**Affiliations:** University College Dublin, Ireland

We would like to thank you for your time and expert peer review of our manuscript. We appreciate your feedback and feel it has improved the quality of the protocol. We have provided a point-by-point response (in italics) indicating how we addressed each concern raised by each reviewer and the changes made. The changes are approved by all authors and highlighted in bold in addition to being tracked changes in the accompanying manuscript.  

Reviewer Comment: In this manuscript, the authors propose a systematic review of the literature on breastfeeding self-efficacy during pregnancy. Overall, the authors have been clear about their goals.

*Response: We thank the reviewer for this positive feedback.*

Reviewer Comment: One suggestion is to expand the section on self-efficacy. Tell readers how it influences health behavior and is super relevant to many aspects of physical and mental health. Then leap to Dennis' adaptation of it to breastfeeding.1 I've always thought that it was relevant.

*Response: We agree and thank the reviewer for this suggestion. We have included this point and expanded on the references to improve the coverage of the review. The text in paragraph 7 of the introduction now reads:* **In general, self-efficacy is positively associated with decision making around health behaviours, both in terms of physical and mental health1-3. Breastfeeding self-efficacy has been identified as a strong modifiable predictor of breastfeeding initiation, duration, and exclusivity 18, 19 . The term stems from Dennis’ adaptation of Bandura’s Social Cognitive Theory on self-efficacy and is described as a woman’s self-belief and confidence in her perceived ability to breastfeed 20, 21** *.*

**1.  Hevey D. Self-efficacy and health behaviour: A review. The Irish Journal of Psychology. 1998;19((2-3)):248–73.**

**2. Sachs-Ericsson N, Medley AN, Kendall-Tackett K, Taylor J. Childhood Abuse and Current Health Problems among Older Adults: The Mediating Role of Self-Efficacy. Psychol Violence. 2011;1(2):106-20.**

**3. Sheeran P, Maki A, Montanaro E, Avishai-Yitshak A, Bryan A, Klein WMP, et al.** **The impact of changing attitudes, norms, and self-efficacy on health-related intentions and behavior: A meta-analysis. Health Psychology. 2016;35(11):1178-88.**

Reviewer comment: I'm not sure if you want to include this, but so much of the literature is about prenatal attachment and breastfeeding. The authors might want to distinguish between the two and offer justification for why they are focusing on self-efficacy.

*Response: We agree that a lot of valuable literature on prenatal attachment and breastfeeding exists. We feel that breastfeeding self-efficacy is under-explored within the prenatal period, which is our rationale for focusing on this area. We agree that clarification would be helpful and have amending the eligibility criteria to exclude prenatal attachment studies [Table 1. Eligibility criteria incorporating the (PCC) framework - Problem, Concept, Context]:*

**Concept: Self-Efficacy - The review will include literature related to breastfeeding self-efficacy and exclude those focusing on self-efficacy of other parenting areas, and studies on prenatal attachment.**

Reviewer comment: Are there a lot of articles looking at self-efficacy in early pregnancy? I would think that that is a fairly novel idea. Would there be enough literature to compare early vs late prenatal self-efficacy?

*Response: Thank you for this observation. We agree that there may not be a lot of articles, which is why we selected a scoping review methodology to map the literature that does exist. We will potentially be able to explore the timing of self-efficacy measurements across the prenatal period, but this will be contingent on what data is identified.*

Reviewer comment: Is the aim to "explore the theoretical underpining" of self-efficacy? That seems different than what was described in the intro. What constructs would the authors explore? It might be good to highlight those if the authors have some ideas about what they are.

*Response:  Thank you for this question, one of our three aims is to **:*** **“identify and describe the theoretical frameworks and influencing factors that underpin prenatal breastfeeding self-efficacy”** [Aims & Objectives] *We plan to look at the way studies have constructed the theories that underpinned their examination of self-efficacy, either in their approach or analysis. We do not have a theoretical lens that we are imposing on our mapping of the literature.*

Reviewer comment: The authors forgot to add "lactation" to their list of data sources. They will find a lot there.

*Response: Thank you for highlighting this. Lactation is included as a truncated form* (lactat*) *in the first line of Table 2.*

Reviewer comment: PubMed is not included in the search? That seems like a key source. It would be good to include it.

*Response: We agree that PubMed is a very useful database. Medline and PubMed cover the same databases and our specialist librarian advised us to use Medline.*

Reviewer comment: I am a bit concerned that much of the "grey literature" would not have the rigor required to be included. Will this literature be subjected to the same criteria or will it be different?

*Response: Thank you for raising this concern. Scoping reviews are useful tools to systematically identify and map the existing evidence. We will not be seeking to evaluate the quality of the evidence as this aligns with the focus of a systematic review and we do not yet have the knowledge of the extent of the literature to undertake such work, hence our need for a scoping review. To address the rigour of study selection and data extraction, a systematic approach is being adopted, including all stages of screening and data extraction conducted independently by at least 2 authors. The characteristics of any grey literature included in the review will be described. However, we agree that more clarity would be helpful and we have amended the text in the data extraction section as follows:* **“The data extraction for grey literature will depend on the nature of the data, following the methods outlined above when applicable and following a qualitative approach when required, extracting themes and quotes from sources.”** *In response to feedback from both reviewers on our treatment of grey literature, we have removed the reference to assessing the level of evidence against the JBI categories in the final paragraph of the data extraction section (see below). We thank the reviewers for highlighting our methodological inconsistency and feel this change aligns the manuscript more fully with scoping review methods.*

**“A draft extraction form is provided in Table 4. A critical appraisal of the evidence will not be conducted as this is beyond the purpose of a scoping review. In its place, the reviewers will assess what level of evidence each individual source comes from against the JBI categories. This will give the scoping review the ability to provide comment on the quality of the evidence informing the state of understanding.”**

Reviewer comment: I'm looking forward to seeing the final review. I think it will be helpful.

*Response: We thank the reviewer again for their feedback and encouragement.*
